# Supplementary material for: Myc-dependent dedifferentiation of Gata6+ epidermal cells resembles reversal of terminal differentiation
Source: Nat Cell Biol. 2023 Sep 21;25(10):1426–38. doi: 10.1038/s41556-023-01234-5 (PMC10567550; doi:10.1038/s41556-023-01234-5)
Supplement: Supplementary file 1 — Reporting Summary [file 41556_2023_1234_MOESM1_ESM.pdf]

nature portfolio

Corresponding author(s): Fiona M Watt

Last updated by author(s): Jul 26, 2023

Reporting Summary

Nature Portfolio wishes to improve the reproducibility of the work that we publish. This form provides structure for consistency and transparency in reporting. For further information on Nature Portfolio policies, see our Editorial Policies and the Editorial Policy Checklist.

Statistics

For all statistical analyses, confirm that the following items are present in the figure legend, table legend, main text, or Methods section.

n/a	Confirmed	
☐	☒	The exact sample size (*n*) for each experimental group/condition, given as a discrete number and unit of measurement
☐	☒	A statement on whether measurements were taken from distinct samples or whether the same sample was measured repeatedly
☐	☒	The statistical test(s) used AND whether they are one- or two-sided<br>*Only common tests should be described solely by name; describe more complex techniques in the Methods section.*
☒	☐	A description of all covariates tested
☐	☒	A description of any assumptions or corrections, such as tests of normality and adjustment for multiple comparisons
☐	☒	A full description of the statistical parameters including central tendency (e.g. means) or other basic estimates (e.g. regression coefficient) AND variation (e.g. standard deviation) or associated estimates of uncertainty (e.g. confidence intervals)
☐	☒	For null hypothesis testing, the test statistic (e.g. *F*, *t*, *r*) with confidence intervals, effect sizes, degrees of freedom and *P* value noted<br>*Give P values as exact values whenever suitable.*
☒	☐	For Bayesian analysis, information on the choice of priors and Markov chain Monte Carlo settings
☒	☐	For hierarchical and complex designs, identification of the appropriate level for tests and full reporting of outcomes
☒	☐	Estimates of effect sizes (e.g. Cohen's *d*, Pearson's *r*), indicating how they were calculated

Our web collection on statistics for biologists contains articles on many of the points above.

Software and code

Policy information about availability of computer code

Data collection	Confocal microscopy: Nikon A1 confocal microscope, Nikon A1R confocal, Nikon A1RMP upright microscope.<br>Atomic Force Microscopy: AFM measurements were carried out using a Bioscope atomic force microscope (Bioscope resolveTM BioAFM, Bruker), coupled with an optical microscope (Nikon Eclipse Ti-U)<br>Flow cytometry: BD FACSAria II cell sorter<br>NanoZoomer 2.0RS Digital Slide Scanner (Hamamatsu Photonics K.K)<br>Sequencing: Illumina HiSeq4000 75 PE
Data analysis	Data and statistical analysis: Excel (Microsoft) (Version 2305 Build 16.0.16501.20074)<br>Data and statistical analysis: Prism (Graphpad version 9.5.1)<br>Image analysis: Fiji or Image J (NIH) (v1.53)<br>Image analysis: NPD viewer software (Hamamatsu, v2.7.43)<br>RNA-seq: Smart-seq2 sequencing data was aligned with STAR (version 2.2), using the STAR index and aligned to GRCm38 reference genome. Gene-specific counts were calculated using featureCounts function from Rsubread (version3.7) with mm10 RefSeq annotation, and analysed with Seurat version4.1.1. Marker genes of each cell cluster/state were outputted for enrichment analysis using fgsea version1.16.0 package in R. Monocle 2 v2.18.0 and Monocle 3 v0.1.3 (pseudotime/trajectory analysis). CellRank (v1.5.1) was used to asses cell dynamics. For most plots we used ggplot2 (v3.3.6).<br>Flow cytometry analysis: FlowJo software (v10.7.2)

For manuscripts utilizing custom algorithms or software that are central to the research but not yet described in published literature, software must be made available to editors and reviewers. We strongly encourage code deposition in a community repository (e.g. GitHub). See the Nature Portfolio guidelines for submitting code & software for further information.

Data

Policy information about availability of data

All manuscripts must include a data availability statement. This statement should provide the following information, where applicable:

- Accession codes, unique identifiers, or web links for publicly available datasets
- A description of any restrictions on data availability
- For clinical datasets or third party data, please ensure that the statement adheres to our policy

The single-cell RNA-sequencing data have been deposited in the Gene Expression Omnibus (GEO) under the accession code GSE174857. The mouse reference genome sequence (GRCm38) was downloaded from Ensembl (http://ensembl.org/Mus_musculus) and used for alignment of the single-cell RNA-seq data.

Human research participants

Policy information about studies involving human research participants and Sex and Gender in Research.

Reporting on sex and gender	N/A
Population characteristics	N/A
Recruitment	N/A
Ethics oversight	N/A

Note that full information on the approval of the study protocol must also be provided in the manuscript.

Field-specific reporting

Please select the one below that is the best fit for your research. If you are not sure, read the appropriate sections before making your selection.

☒ Life sciences ☐ Behavioural & social sciences ☐ Ecological, evolutionary & environmental sciences

For a reference copy of the document with all sections, see nature.com/documents/nr-reporting-summary-flat.pdf

Life sciences study design

All studies must disclose on these points even when the disclosure is negative.

Sample size	No statistical methods were used to predetermine sample sizes. The sample size was determined based on previous experience with preliminary experiments and previous studies (doi.org/10.1016/j.celrep.2018.09.059; doi.org/10.1038/ncb3532; doi.org/10.1038/s41586-020-2555-7). In accordance with local animal ethics, the experiments were designed to use the smallest number of mice needed to obtain the requested data. All n values are clearly stated in the Figure legends.
Data exclusions	No exclusion was applied
Replication	All experiments were repeated at least three independent times with independent samples, unless otherwise specified. Precise n values are stated in the Figure legends. All attempts at replication were successful.
Randomization	Based on their genotype, the mice were allocated randomly to experimental groups. There was no allocation into experimental groups in other experiments, thus randomization is not relevant beyond mouse experiments in this study.
Blinding	The investigators were not blinded to allocation during experiments and outcome assessment

Reporting for specific materials, systems and methods

We require information from authors about some types of materials, experimental systems and methods used in many studies. Here, indicate whether each material, system or method listed is relevant to your study. If you are not sure if a list item applies to your research, read the appropriate section before selecting a response.

Materials & experimental systems

n/a	Involved in the study
☐	☒ Antibodies
☐	☒ Eukaryotic cell lines
☒	☐ Palaeontology and archaeology
☐	☒ Animals and other organisms
☒	☐ Clinical data
☒	☐ Dual use research of concern

Methods

n/a	Involved in the study
☒	☐ ChIP-seq
☐	☒ Flow cytometry
☒	☐ MRI-based neuroimaging

Antibodies

Antibodies used

Krt14 (1:1,000, LL002 clone, Abeam ab7800 and 1:1,000, Covance SIG-3476); ltga6 (1:200, GoH3 clone, eBioscience 14-0495-81); Myc (1:100, Abeam ab32072); Ki67 (1:50, Novus BIologicals, NB600-1252); Lrig1 (1:200, R&D Systems AF3688); lnvolucrin (1:500, ERLI-3 clone, in-house); CD45 (1:200, 30-F11 clone, eBioscience 14-0451-82); pMLC (1:100, Cell Signaling, #3674); YAP (1:200, Cell Signaling, #14074); MAL (1:200, Proteintech, 21166-1-AP); anti-CD45 APC (1:1500, eBioscience, #17-0451-82); anti-CD49f (1:500, ltga6, Biolegend, clone GoH3, #313611). Secondary antibodies conjugated to Alexa Fluor 488 or 647 (1:500) were purchased from lnvitrogen: Donkey anti-Rabbit IgG (H+L), Alexa Fluor 647, A32795; Donkey anti-Rat IgG (H+L), Alexa Fluor 647, A48272; Donkey anti-Goat IgG (H+L), Alexa Fluor 488, A48272; Goat anti-Chicken IgG (H+L), Alexa Fluor 488, A21449; Goat anti-Rabbit IgG (H+L), Alexa Fluor 488, A11034

Validation

All antibodies were published and validated in previous studies. Validation statements can be found on the manufacturer's website.
Krt14. https://www.abcam.com/cytokeratin-14-antibody-11002-ab7800.html
ltga6. https://www.thermofisher.com/antibody/product/CD49f-lntegrin-alpha-6-Antibody-done-eBioGoH3-GoH3-Monoclonal/14-0495-82
Myc. https://www.abcam.com/c-myc-antibody-y69-ab32072.html
Ki67. https://www.novusbio.com/products/ki67-mki67-antibody-sp6_nb600-1252
Lrig1. https://www.rndsystems.com/products/mouse-lrig1-anti body_af3688
lnvolucrin. doi: 10.1083/jcb.200706187
CD45. https://www.thermofisher.com/antibody/product/CD45-Antibody-clone-30-F11-Monoclonal/14-0451-82
pMLC. https://www.cellsignal.co.uk/products/primary-antibodies/phospho-myosin-light-chain-2-thr18-ser19-antibody/3674
YAP. https://www.cellsignal.com/products/primary-antibodies/yap-d8h1x-xp-rabbit-mab/14074
MAL. https://www.ptglab.com/products/MKL1-Antibody-21166-1-AP.htm
CD45 APC. https://www.thermofisher.com/antibody/product/CD45-Antibody-clone-30-F11-Monoclonal/17-0451-82
CD49f. https://www.biolegend.com/en-us/search-results/pe-anti-human-mouse-cd49f-antibody-4108
Donkey anti-Rabbit, Alexa Fluor 647. https://www.thermofisher.com/antibody/product/Donkey-anti-Rabbit-IgG-H-L-Highly-Cross-Adsorbed-Secondary-Antibody-Polyclonal/A32795
Donkey anti-Rat, Alexa Fluor 647, A48272. https://www.thermofisher.com/antibody/product/Donkey-anti-Rat-IgG-H-L-Highly-Cross-Adsorbed-Secondary-Antibody-Polyclonal/A48272
Donkey anti-Goat, Alexa Fluor 488. https://www.thermofisher.com/antibody/product/Donkey-anti-Goat-IgG-H-L-Cross-Adsorbed-Secondary-Antibody-Polyclonal/A-11055
Goat anti-Chicken, Alexa Fluor 488. https://www.thermofisher.com/antibody/product/Goat-anti-Chicken-IgY-H-L-Secondary-Antibody-Polyclonal/A-21449
https://www.thermofisher.com/antibody/product/Goat-anti-Rabbit-IgG-H-L-Highly-Cross-Adsorbed-Secondary-Antibody-Polyclonal/A-11034

Eukaryotic cell lines

Policy information about cell lines and Sex and Gender in Research

Cell line source(s)

- Spontaneously immortalised keratinocytes isolated from K14MycER transgenic mouse founder line 2184C.1 (doi.org/10.1016/S0960-9822(01)00154-3).
- 3T3-J2 cells were originally obtained from Dr. James Rheinwald (Department of Dermatology, Harvard Skin Research Centre, USA).

Authentication

The identity of the keratinocytes isolated from K14MycER transgenic mice was validated by activation of Myc on Tamoxifen treatment. Both keratinocytes and 3T3-J2 cells grew and showed the expected morphology. No additional specific authentication was performed.

Mycoplasma contamination

All cell stocks were routinely tested for mycoplasma contamination and found to be negative.

Commonly misidentified lines
(See ICLAC register)

No commonly misidentified cell lines were used in the study

Animals and other research organisms

Policy information about studies involving animals; ARRIVE guidelines recommended for reporting animal research, and Sex and Gender in Research

Laboratory animals	Mus musculus. This study includes adult mice (8-12 weeks). The following strains were used: Rosa26-fl/STOP/fltdTomato43, CAGGS eGFP, Gata6EGFPCreERT2, c-Myc fl/fl mice.
Wild animals	No wild animals were used in this study.
Reporting on sex	Experiments were carried out with male and female mice. No gender-specific differences were observed.
Field-collected samples	No field-collected samples were used in the study.
Ethics oversight	All mouse procedures were subjected to local ethical approval at King's College London (UK) and performed under a UK Government Home Office (PP70/8474 or PP0313918).

Note that full information on the approval of the study protocol must also be provided in the manuscript.

Flow Cytometry

Plots

Confirm that:

☒ The axis labels state the marker and fluorochrome used (e.g. CD4-FITC).

☒ The axis scales are clearly visible. Include numbers along axes only for bottom left plot of group (a 'group' is an analysis of identical markers).

☒ All plots are contour plots with outliers or pseudocolor plots.

☒ A numerical value for number of cells or percentage (with statistics) is provided.

Methodology

Sample preparation	Single keratinocytes from wounded and unwounded skin of a Gata6EFGPCreERT2 Rosa26-fl/STOP/fl-tdTomato43 mouse were isolated by flow sorting, and labelled with anti-CD45 and anti-CD49f (Itga6). The samples were buffered with GFP+ epidermal cells from CAGGS eGFP mice, in which GFP is expressed in all cells via the CMV-b-actin promoter. GFP+ epidermal cells were subsequently removed by sorting.
Instrument	Cell sorting was performed on the BD FACSAria II cell sorter.
Software	Data were analysed using FlowJo software.
Cell population abundance	Sorted samples were >95% pure. A small fraction of fibroblasts was discarded from the analysis.
Gating strategy	Epidermal cells were gated on FSC/SSC- area and width, live (DAPI), GFP- CD45-, tdTomato+ltg6-, tdTomato+ltg6+, tdTomato-ltg6-, and tdTomato-ltg6+.

☒ Tick this box to confirm that a figure exemplifying the gating strategy is provided in the Supplementary Information.
